# Understanding quality-of-life patterns in long COVID: How Symptoms and socioeconomic conditions shape patient wellbeing

**DOI:** 10.1371/journal.pone.0347743

**Published:** 2026-04-20

**Authors:** Esther Ortega-Martin, Javier Alvarez-Galvez

**Affiliations:** 1 Department of General Economy (Health Sociology area), Faculty of Nursing and Physiotherapy, University of Cadiz, Cadiz, Spain; 2 Computational Social Science DataLab, University Institute for Sustainable Social Development, University of Cádiz, Jerez de la Frontera, Spain; University of Diyala College of Medicine, IRAQ

## Abstract

**Objective:**

To characterize the heterogeneity of Long COVID (LC) by identifying distinct patient profiles based on symptoms and quality of life (QoL), and to examine the sociodemographic and clinical predictors associated with these profiles.

**Study design:**

A cross-sectional observational study was conducted.

**Methods:**

We recruited 363 patients with LC in Spain via an online survey. Symptom patterns were identified through latent class analysis of 15 binary symptoms. QoL was assessed with the patient-derived LC-6D-QoL across six dimensions, and cluster analysis defined QoL subgroups. Logistic regression was applied to examine clinical and sociodemographic predictors of QoL profiles.

**Results:**

Two symptom profiles emerged: a low-burden profile, dominated by fatigue and cognitive problems, and a high-burden profile with multisystem involvement. QoL clustered into three profiles—high, middle, and low QoL—with more than half of participants in the low QoL group. Symptom burden and employment status were the strongest predictors of poor QoL, whereas age, sex, education, and income showed limited associations. Social support was more frequently reported among participants with low QoL.

**Conclusions:**

LC is characterized by distinct clinical and QoL profiles, with strong interactions between multisystem symptom burden and social determinants. Identifying patients at greatest risk of poor QoL can inform stratified interventions and integrated policies that combine medical care, psychosocial support, and workplace reintegration.

## Introduction

Long COVID (LC) —also referred to as post-COVID-19 condition (PCC) [1,2]— is a multisystem condition recognized by the World Health Organization (WHO) as the persistence or recurrence of symptoms for at least three months after acute SARS-CoV-2 infection [3]. In 2024, the National Academies of Sciences, Engineering, and Medicine (NASEM) defined LC as a chronic condition lasting ≥3 months, with continuous, relapsing-remitting, or progressive courses affecting multiple organ systems [4]. However, prevalence estimates vary depending on definition and methods, from around 6% to 15% [5,6].

Symptoms are heterogeneous and span multiple systems, including fatigue, dyspnoea, chest pain, cough, post-exertional malaise, and cognitive disturbances such as “brain fog” [7–12]. In addition, psychological manifestations, such as anxiety, depression, and post-traumatic stress [10,13,14], are also common [10,13,14]. This variability complicates diagnosis and management, contributing to a fluctuating and often disabling clinical course. Consequently, LC patients report limitations in daily activities, multidimensional QoL impairments, and substantial economic difficulties [15–17]. Furthermore, recent studies have highlighted symptom clusters linked to poor QoL [18,19], as well as sociodemographic predictors such as sex, income, and employment status [20,21]. However, most research has examined symptoms, sociodemographic factors, and QoL separately, without integrating them into patient-centred profiles.

Some studies have attempted to characterize LC through symptom-based classifications. For example, Wong et al. [22] identified three phenotypes among 1,344 patients—fatigue and dyspnoea, anxiety and depression, and a combined fatigue–dyspnoea–anxiety–depression class—the latter associated with the lowest quality of life (QoL). Similarly, Vartanian et al. [23] described four subgroups among 634 participants, ranging from a few lingering symptoms to co-occurring physical, cognitive, and mental health challenges. While these studies provide valuable insights into symptom heterogeneity, they do not integrate multidimensional QoL outcomes or sociodemographic determinants, which limits their applicability for patient-centred care and policy planning.

QoL is a multidimensional construct encompassing physical health, autonomy, mental health, social relationships, and economic and occupational aspects—all of which have been shown to be disrupted in LC [15,24]. Although both generic instruments, such as the EQ-5D-5L, and disease-specific tools, such as the PAC-19QoL, have been employed, existing research remains fragmented and rarely identifies distinct QoL profiles or their predictors.

In this study, we address this gap by applying latent class analysis (LCA) to characterize symptom profiles and cluster analysis to group patients according to their QoL domains, using data from a national cohort of patients with LC in Spain. We then examine the sociodemographic predictors of these profiles. This contribution lies in developing a patient-centred typology that links symptom burden and lived experience to functional outcomes, thereby providing a framework for more precise clinical care, tailored rehabilitation strategies, and evidence-based policy planning.

The main objective of this study was to characterize distinct QoL profiles among patients with LC by integrating symptomatology and sociodemographic factors, to better understand the heterogeneity of their lived experiences and functional outcomes. To achieve this goal, the study addressed the following specific objectives: (i) to identify symptom profiles in patients with LC using latent class analysis; (ii) to characterize subgroups of patients according to their QoL using cluster analysis; and (iii) to examine the sociodemographic and clinical factors associated with membership in each QoL profile through multinomial logistic regression models.

## Materials and methods

### Design and setting

A cross-sectional observational study was conducted between 20 March and 25 June 2025 using a self-administered online survey distributed through patient associations, social media, and collaborating healthcare centres in Spain. Inclusion criteria were age ≥ 18 years; residence in Spain; ability to complete the survey in Spanish. Exclusion criteria: incomplete core variables (symptoms or QoL) or self-report of acute COVID-19 (<3 months).

### Measures and variables

The survey captured sociodemographic variables (age, sex, education, household income, employment), and clinical variables (time since acute infection, acute-phase hospitalization, recognized disability status).

QoL was evaluated using the Long COVID Six-Dimensional Quality of Life instrument (LC-6D-QoL) [25], which assesses general, physical, mental, functional, social and economic domains. Items were rated on a five-point Likert scale, with higher scores indicating better QoL. The LC-6D-QoL was developed through a prior Delphi study establishing its content validity [25]. For this study, the instrument was administered with minor adaptations: two items (sexual/romantic life and workplace adaptations) were excluded due to systematic non-response and partial survey dropouts observed during piloting.

Symptom profiles were captured as binary variables (presence/absence) for 15 domains frequently reported in LC literature [1,26–29]: fatigue, dyspnoea, cognitive impairment, neuropsychological symptoms (anxiety/depression/PTSD), cough, sleep disturbances, chest pain, digestive symptoms, fever, palpitations, dizziness, skin manifestations, visual disturbances, musculoskeletal pain, and dysautonomia.

Most variables had complete data. Missingness was limited to household income (20.4%), municipality size (13.5%), and employment category (3.6%). Little’s MCAR test indicated that data were not missing completely at random (χ² = 24.32, df = 9, p = 0.004). Given the exploratory nature of the study and the low proportion of missingness in key variables, a complete-case analysis was deemed appropriate. No imputation was performed; analyses were based on available cases, and variables with missing data were excluded from specific analyses.

### Data analysis

Latent class analysis (LCA) was used to identify symptom patterns in patients with LC using 15 binary symptoms. Using the poLCA package in R (version 4.5.0) [30], we estimated models with 2–5 classes, each with 20 random starts to mitigate convergence to local maxima. Model selection prioritized the Bayesian Information Criterion (BIC) [31]; we also examined AIC and the likelihood-ratio statistic (G²). Classification quality was summarized by normalized entropy and average posterior probabilities within assigned classes [32]; individuals were assigned to the class with the highest posterior probability (modal assignment). To aid interpretation, we visualized class-specific conditional endorsement probabilities using heatmaps and probability/profile plots.

Quality-of-life profiles were derived from the six LC-6D-QoL domain scores using k-means clustering. Candidate solutions with two to four clusters were evaluated, and the optimal number was determined by the silhouette and elbow criteria. To assess robustness, we performed a sensitivity analysis using hierarchical clustering (Ward’s method), which showed strong agreement with the k-means solution (Cramer’s V = 0.844, p < 0.001). Clusters were described by their centroids and labelled as Low, Medium, and High QoL (reflecting increasing QoL). To assess the internal validity of the QoL clusters, we computed a global QoL score as the mean of the 14 LC-6D-QoL items. Mean differences across clusters were examined using one-way ANOVA. Homogeneity of variances was evaluated with Levene’s test, and post hoc comparisons were conducted with Tukey or Games–Howell tests depending on variance assumptions. Effect sizes were reported as η² with 95% confidence intervals. Clustering analyses were conducted in IBM SPSS Statistics v29.

Finally, multinomial logistic regression was employed to examine clinical and sociodemographic predictors of QoL profiles, using cluster membership as the dependent variable. Independent variables included symptom-burden class, age, sex, education, income, and employment status. Multicollinearity was assessed using Variance Inflation Factors (all VIFs < 2.5), indicating no concerning collinearity. Model fit was evaluated using likelihood-ratio tests and Nagelkerke’s pseudo-R². Given the exploratory nature of this study, we did not adjust for multiple comparisons; results should therefore be interpreted focusing on effect sizes and consistency across models rather than on isolated p-values. Regression analyses were conducted using the nnet package [33] in R (version 4.5.0).

### Ethical statement

The study protocol was approved by the Research Ethics Committee of Cádiz (PEIBA 0659-N-23) and conducted in accordance with the Declaration of Helsinki and the European General Data Protection Regulation (GDPR). Data were collected using LimeSurvey, a secure online platform compliant with EU data protection standards. Participation was voluntary and anonymous. Before accessing the questionnaire, all participants received study information and provided electronic informed consent.

## Results

A total of 363 participants were included. Most were aged 45–65 years (64.7%), followed by 30–44 years (21.8%), ≥ 65 years (12.9%), and 18–29 years (0.6%). Women accounted for 79.6% of the sample. A recognized disability was reported by 30.6%. Educational attainment was diverse, ranging from primary (10.7%) to postgraduate studies (19.8%), with secondary (36.6%) and university degrees (32.2%) most frequent. Household income varied widely, and 20.4% preferred not to disclose. Participants resided in municipalities spanning rural (<2,000 inhabitants) to large metropolitan areas (>1,000,000 inhabitants) (See Table 1).

**Table 1:** Baseline sociodemographic and clinical characteristics of participants with LC.

Variable	Categories/ Descriptive Statistics	n	%
**Sociodemographic characteristics**			
**Age categories**	Young adults (18–29)	2	0.6
	Early middle-aged adults (30–44)	79	21.8
	Late middle-aged adults (45–65)	235	64.7
	Older adults (65+)	47	12.9
**Sex**	Male	74	20.4
	Female	289	79.6
**Educational level**	Primary education/ Basic School	39	10.7
	Secondary education (High School, Vocational Training)	133	36.6
	University degree (Bachelor’s, Diploma, Licentiate)	117	32.2
	Postgraduate (Master’s, PhD)	72	19.8
	Prefer not to answer	2	0.6
**Monthly household net income**	≤ €600	24	6.6
	€601 – €900	28	7.7
	€901 – €1,200	53	14.6
	€1,201 – €1,800	58	16.0
	€1,801 – €2,400	52	14.3
	€2,401 – €3,000	32	8.8
	€3,001 – €4,500	27	7.4
	€4,501 – €6,000	9	2.5
	More than €6,000	6	1.7
	Prefer not to answer	74	20.4
**Municipality population size**	≤ 2,000 inhabitants	34	9.4
	2,001–10,000 inhabitants	44	12.1
	10,001–50,000 inhabitants	51	14.0
	50,001–100,000 inhabitants	28	7.7
	100,001–400,000 inhabitants	76	20.9
	400,001–1,000,000 inhabitants	32	8.8
	More than 1,000,000 inhabitants	49	13.5
	Not specified	49	13.5
**Clinical characteristics and healthcare utilization**			
**Disability status**	Yes	111	30.6
	No	252	69.4
**Time with LC**	6 months to <1 year	1	0.3
	1 year to <2 years	12	3.3
	2 years to <3 years	49	13.5
	3 years or more	301	82.9
**Hospitalization during acute COVID-19**	Prefer not to answer	5	1.4
	Yes, hospital ward	55	15.2
	Yes, ICU	17	4.7
	No, but received outpatient care	157	43.3
	No, managed at home without medical care	129	35.5

Regarding clinical characteristics, most participants had lived with LC for over three years (82.9%), indicating a prolonged disease course. Nearly one-third reported a recognized disability (30.6%). Around one in five had been hospitalized during the acute infection—15.2% in hospital wards and 4.7% in intensive care units—while the majority managed COVID-19 at home or through outpatient care.

**Fig 1:**
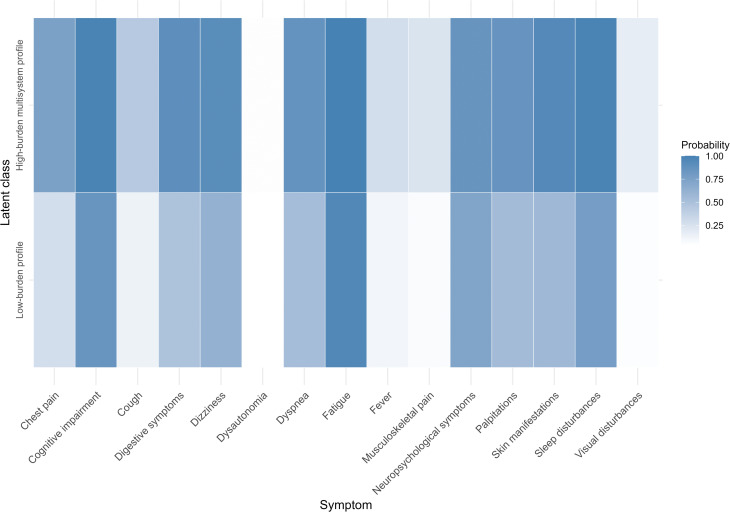
Symptoms probabilities across latent classes. Conditional probabilities of reporting each of the 15 symptoms for the low-burden profile (n = 133) and the high-burden multisystem profile (n = 230). Darker colors indicate higher probabilities.

Latent class analysis identified a two-class solution as the best fit. Model fit indices indicated that the two-class solution provided the best balance of fit and parsimony (AIC = 4691.8; BIC = 4812.5; entropy = 0.65), with models including three or more classes showing no meaningful improvement. The *low-burden profile*, corresponding to Class 1 (n = 133; 36.6%), showed generally low symptom endorsement and was defined by fatigue, cognitive difficulties, neuropsychological complaints and sleep disturbance, with only moderate probabilities of dyspnoea, palpitations, dizziness and skin manifestations. In contrast, *the high-burden multisystem profile*, corresponding to Class 2 (n = 230; 63.4%), displayed consistently high endorsement across domains, with particularly elevated probabilities of dyspnoea, cognitive impairment, sleep disturbance, palpitations, dizziness and digestive symptoms. The symptom endorsement probabilities clearly distinguished these two groups, with one showing consistently low levels and the other widespread multisystem involvement (See Fig 1).

**Fig 2:**
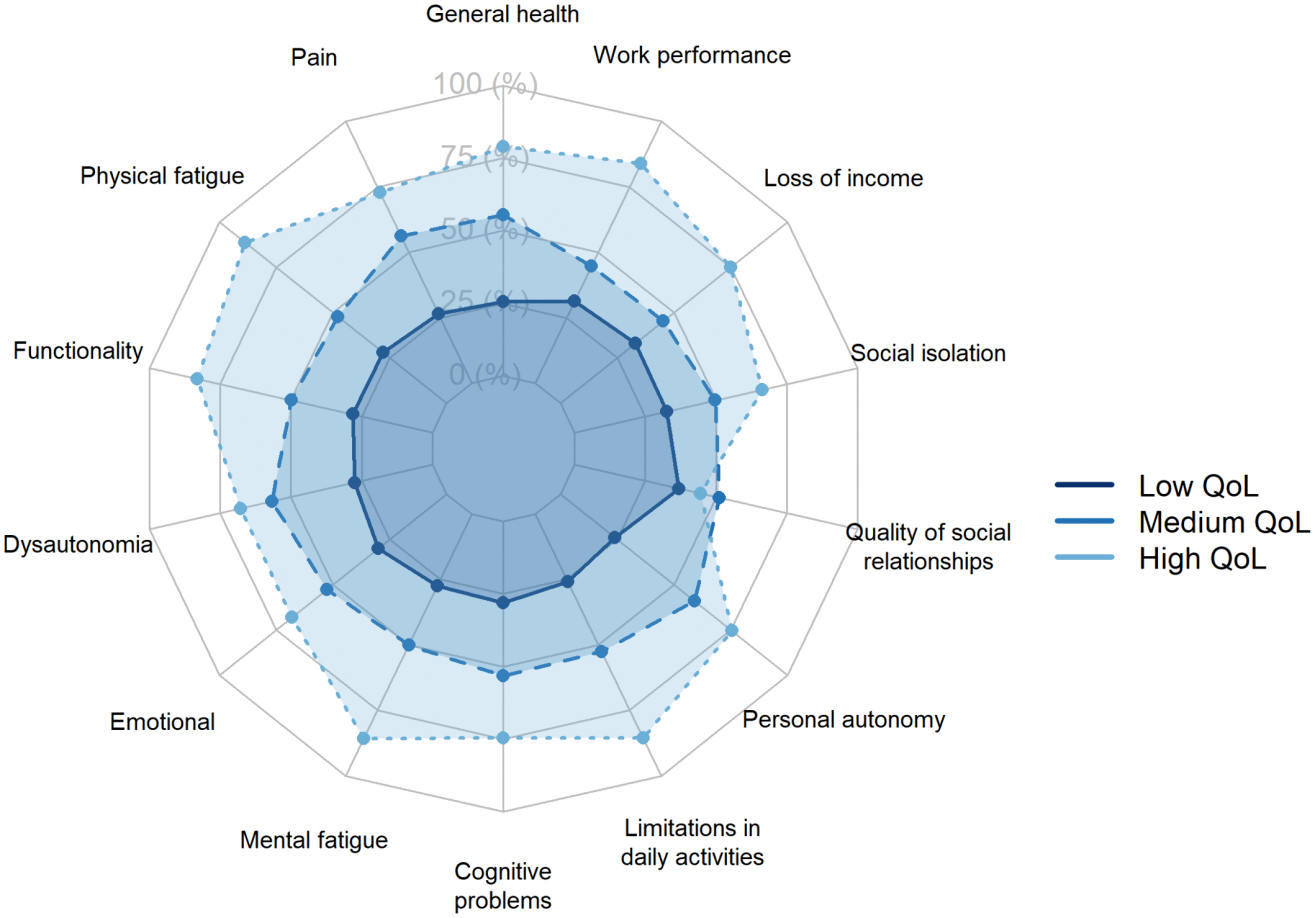
Radar plot of QoL domains by profile in patients with LC. Radar plot showing mean standardized scores for the three QoL clusters: High QoL (least impaired), Medium QoL, and Low QoL (most impaired). Higher values indicate better QoL.

The analysis of QoL domains revealed three distinct profiles: High QoL (least impaired) (n = 48; 13.2%), Medium QoL (n = 124; 34.2%), and Low QoL (most impaired) (n = 191; 52.6%). These profiles were derived from clustering of the LC-6D-QoL domains, which encompassed physical health including pain, mental health, daily functioning and health perception, autonomy, cognitive functioning, social relationships and support, and economic/occupational aspects. The robustness of the three-cluster solution was confirmed by hierarchical clustering (Ward’s method), which showed strong agreement with the k-means solution (Cramer’s V = 0.844, p < 0.001). Centroid patterns showed a graded severity across clusters. To assess internal validity, we compared the clusters on a global QoL index derived as the mean of all LC-6D-QoL items. Differences were large and statistically significant (ANOVA, F (2,360) =737.95, p < 0.001, η² = 0.804, 95% CI 0.771–0.828). Post hoc Games–Howell tests confirmed significant pairwise differences between all clusters (p < 0.001). Mean standardized scores were −0.77 (SD = 0.41) in the Low QoL group, 0.50 (SD = 0.42) in the Medium QoL group, and 1.76 (SD = 0.60) in the High QoL group. Participants in the High QoL group reported better perceived health, lower pain and fatigue, fewer limitations, and preserved autonomy. In contrast, the Medium QoL cluster displayed intermediate impairment, while the Low QoL cluster exhibited uniformly poor scores across domains, particularly in daily functioning, cognition, and occupational performance. Social support showed the smallest differentiation between clusters. Differences across clusters were significant, with all comparisons meeting conventional thresholds of statistical significance, and the three-class solution converged within 15 iterations (Fig 2).

Finally, three multinomial regression models were estimated to examine predictors of QoL cluster membership, taking High QoL as the reference category (See Table 2). Model 1, which included all predictors, showed no significant associations for age, sex, education, or household income, while symptom burden, identified by LCA, was strongly associated with poorer outcomes. In Model 2, restricted to demographic and clinical factors, symptom burden again emerged as the main predictor, and age showed a borderline and small association with medium QoL (OR=1.03; 95% CI 1.00–1.06; p = 0.051), whereas sex was not significant. In Model 3, which incorporated socioeconomic and clinical variables, the effect of symptom burden was confirmed (OR=17.27; 95% CI 6.42–46.43; p < 0.001 for the comparison between Low QoL and High QoL). Employment status was independently associated with low QoL: compared with active workers, unemployed participants had 86% lower odds of low QoL (OR=0.14; 95% CI 0.02–0.94; p = 0.043), and those on sick leave or incapacity showed 95% lower odds (OR=0.05; 95% CI 0.01–0.42; p = 0.006). Although Model 1 achieved slightly higher overall and balanced accuracy in classifying QoL groups, Model 3 provided the most parsimonious fit (AIC = 473; BIC = 539), with comparable explanatory power. Furthermore, Model 3 also improved the identification of participants with low QoL, a clinically relevant subgroup. Taken together, these analyses identify symptom burden as the strongest and most consistent predictor of poorer QoL, a borderline effect of age for medium QoL, and a distinct association with employment status once socioeconomic factors were considered.

**Table 2:** Multinomial logistic regressions of predictors of QoL cluster membership.

Predictor	Variable categories	MODEL 1		MODEL 2		MODEL 3	
		Medium QoL OR (95% CI)	Low QoL OR (95% CI)	Medium QoL OR (95% CI)	Low QoL OR (95% CI)	Medium QoL OR (95% CI)	Low QoL OR (95% CI)
Age (years)	Continuous	1.01 (0.97–1.05)	1.04 (0.98–1.11)	1.03 (1.00–1.06)	1.04 (1.00–1.09)		
Sex	Male	1.00 (ref.)	1.00 (ref.)	1.00 (ref.)	1.00 (ref.)		
	Female	0.59 (0.28–1.23)	1.12 (0.36–3.50)	0.75 (0.41–1.35)	0.98 (0.42–2.28)		
LCA	Low-burden profile	1.00 (ref.)	1.00 (ref.)	1.00 (ref)	1.00 (ref)	1.00 (ref)	1.00 (ref)
	High-burden multisystem profile	3.42 (1.84–6.36) ***	18.63 (6.74–51.50) ***	4.01 (2.41–6.69) ***	11.57 (5.51–24.28) ***	3.61 (1.96–6.68) ***	17.27 (6.42–46.43) ***
Employment	Work	1.00 (ref.)	1.00 (ref.)			1.00 (ref.)	1.00 (ref.)
	Retired	0.95 (0.41–2.22)	0.34 (0.09–1.24)			1.01 (0.45–2.26)	0.48 (0.15–1.57)
	Unemployed	0.49 (0.20–1.16)	0.18 (0.04–0.82)**			0.50 (0.21–1.19)	0.19 (0.04–0.81)*
	Sick leave/permanent work incapacity	0.29 (0.13–0.63) **	0.02 (0.00–0.21) ***			0.30 (0.14–0.64) **	0.03 (0.00–0.24) ***
Education	High education	1.00 (ref.)	1.00 (ref.)			1.00 (ref.)	1.00 (ref.)
	Medium education	0.58 (0.29–1.14)	0.86 (0.30–2.48)			0.63 (0.32–1.24)	0.83 (0.30–2.32)
	Low education	1.24 (0.47–3.32)	1.63 (0.35–7.53)			1.53 (0.59–3.93)	1.67 (0.37–7.50)
Household income	High income	1.00 (ref.)	1.00 (ref.)			1.00 (ref.)	1.00 (ref.)
	Medium income	0.73 (0.36–1.46)	1.03 (0.38–2.81)			0.74 (0.37–1.47)	0.98 (0.36–2.66)
	Low income	0.78 (0.31–1.97)	0.19 (0.02–2.03)			0.74 (0.29–1.85)	0.17 (0.02–1.74)

Note: OR = odds ratio; CI = confidence interval; ref. = reference category. p < 0.05 (*), p < 0.01 (**), p < 0.001 (***). Nagelkerke R² = 0.379 (Model 1), 0.199 (Model 2), 0.367 (Model 3).

The regression models showed moderate-to-good explanatory power. The full Model 1 explained 37.9% of the variance in QoL (Nagelkerke R² = 0.379), followed by Model 3 (socioeconomic) with 36.7% (R² = 0.367), and Model 2 (reduced) with 19.9% (R² = 0.199).

## Discussion

The present study highlights the clinical heterogeneity of LC, marked impairments in QoL, and the interplay of clinical and social determinants. First, symptoms clustered into two intensity-based profiles: a low-burden profile centred on fatigue and cognitive problems, and a high-burden profile with multisystem involvement. Second, QoL analysis revealed three levels of impairment— High QoL (least impaired), Medium QoL, and Low QoL (most impaired)—with more than half of participants in the most affected group (low QoL). Third, multinomial models indicated that belonging to the high-burden symptom class was strongly associated with lower QoL, and employment status further differentiated risk; by contrast, age, sex, education, and household income were not significantly associated.

The sample consisted predominantly of women and middle-aged participants, consistent with previous reports [34,35]. Given this imbalance, sex-related differences in QoL were not statistically significant after accounting for symptom burden, likely reflecting the small proportion of men in the sample. Nonetheless, multicentre studies such as ORCHESTRA have reported poorer outcomes among women [36]. Similarly, age showed modest and inconsistent associations with QoL, suggesting that impairments affected participants across all age groups rather than being confined to older adults —a finding that contrasts with studies reporting slower recovery among older individuals [37,38]. In terms of socioeconomic factors, education and income were not significant predictors of QoL. In contrast, studies from the USA and the UK have reported clear socioeconomic gradients in LC prevalence and recovery [39,40]. Such divergence may be partly explained, by the Spanish welfare context, where universal healthcare and stronger social protection systems likely attenuate the impact of socioeconomic inequalities on long-term outcomes.

In line with previous studies, a higher symptom burden was associated with lower QoL and greater likelihood of work incapacity, reinforcing the link between multisystem involvement and functional limitations in daily life [41,42]. Similarly, evidence from European cohorts has reported persistent declines in multiple QoL domains, including physical, psychological, and social functioning [15,43]. Moreover, the pattern observed here was primarily defined by overall symptom intensity and the number of systems affected, rather than by specific symptom types. Regarding QoL, Giszas et al. [44] identified two subgroups—preserved and markedly impaired QoL. By modelling three QoL clusters, the analysis captured an intermediate group that helps refine previous dichotomous classifications of LC-related impairment. Finally, although female sex has been linked to worse QoL in other studies, this association was not confirmed after adjusting for symptom burden.

Employment status was associated with QoL, showing an unexpected pattern. Participants who remain employed reported poorer outcomes than those on sick leave or with permanent incapacity, who showed comparatively better QoL. Given the cross-sectional design, these associations cannot be interpreted causally. This may reflect the physical and cognitive strain of maintaining work activities despite persistent symptoms, which can exacerbate fatigue and compromise overall wellbeing [45]. In this study, cognitive dysfunction—particularly difficulties with attention, memory, and mental speed—was common and appeared to contribute to reduced work-related QoL. This aligns with earlier findings reporting reduced work capacity, job disruptions, and prolonged absences from work [45,46]. These findings underline the need for integrated rehabilitation and flexible return-to-work programmes embedded within coordinated public health and social policies. Workplace accommodations—such as graded return-to-work, flexible scheduling, and task modification for cognitive dysfunction and fatigue—should be combined with disability certification processes that recognize the fluctuating nature of LC [47]. Early intervention is critical, as prolonged absence reduces the likelihood of successful return to work [47]. Together, these measures can help restore functional capacity and support sustained labour participation.

Consistent with research on chronic conditions and LC, social support may mitigate the impact of symptom burden and functional compromise [48,49]. Participants in the High QoL group reported lower perceived social support compared with the other groups. This pattern may reflect a lower need for external help among individuals who have regained greater autonomy and stability, or a shift towards more independent coping strategies after prolonged illness.

This study integrates symptom clustering and QoL profiling within the same study population, linking both through regression models. While previous studies have examined symptom clusters [50,51] or QoL profiles separately [44], this analysis offers a combined perspective that shows how variability in symptom patterns is reflected in different QoL profiles. The use of the LC-6D-QoL extends previous research by incorporating patient-derived domains such as autonomy, dysautonomia, and health perception, which are rarely included in LC studies.

The findings suggest that stratifying patients by both symptom and QoL profiles could help optimize healthcare resources and guide tailored interventions, informing the planning of integrated health and social care programmes at the population level. Specifically, this information can guide the design of accessible, tiered care pathways that ensure all patients receive support proportionate to their needs, regardless of their symptom or QoL profile. The multidimensional impact—spanning health, work, and social domains—underscores the need for integrated strategies that combine medical care, occupational rehabilitation, and social protection. The relationship between work ability and QoL is complex: poorer health can limit work participation, while difficulties maintaining employment may, in turn, worsen overall wellbeing. This complexity highlights the need for longitudinal research to disentangle these dynamics and to inform effective reintegration policies [47].

Recruitment in Spain limits the generalizability of the findings, as cultural factors, healthcare organization, and social protection systems may differ across countries and influence patients’ experiences and reported QoL. Our sample was predominantly female (≈80%) and recruited through online platforms and patient associations, which may introduce selection bias toward more chronic and severe cases. Indeed, most participants (83%) had LC for over three years. Therefore, findings may be more representative of persistent LC phenotypes than early or recovering cases, and caution is warranted when generalizing to broader populations. This cross-sectional design means we cannot draw causal conclusions about the relationships between symptoms, employment, and quality of life. The analyses were exploratory and did not include a broader set of covariates; therefore, the findings should be considered preliminary and hypothesis-generating rather than confirmatory. Although developed through a rigorous Delphi study, the LC-6D-QoL has not yet undergone full psychometric validation. This will require a larger, demographically balanced sample—particularly with greater male representation. Additionally, two items (sexual/romantic life and workplace adaptations) from the LC-6D-QoL were excluded due to applicability issues, slightly reducing conceptual coverage but improving data completeness. Nevertheless, the integration of latent symptom classes and QoL clusters within a single study population provides a comprehensive characterization of LC. Finally, the use of the LC-6D-QoL ensured that the evaluated domains reflected patient-relevant outcomes and included aspects often overlooked in generic QoL instruments.

Future work should assess the longitudinal stability of these profiles and replicate findings in larger, more diverse cohorts. Regarding the LC-6D-QoL, future validation studies should reincorporate the two excluded items (affective-sexual life and workplace adaptations) to assess whether a 16-item version yields improved psychometric properties, particularly in domains currently relying on two items that showed lower reliability. It will also be important to test tailored interventions across different patient profiles, addressing specific needs such as cognitive rehabilitation, workplace support, and psychological care. Integrating clinical, biological, and patient-reported data could improve understanding of the mechanisms driving LC and inform the development of more personalized rehabilitation strategies and policy responses.

## Conclusion

This study identified two symptom profiles of LC defined by differences in overall symptom burden, as well as three distinct QoL profiles reflecting varying degrees of multidimensional deterioration. The analyses showed that overall symptom burden and employment status were the main factors associated with patient stratification. These findings provide a framework for understanding heterogeneity in LC and support multidisciplinary responses, underscoring the relevance of both clinical and social dimensions in the long-term follow-up of affected individuals.
